# Four-dot masking in monoptic and dichoptic viewing

**DOI:** 10.1038/s41598-020-67922-6

**Published:** 2020-07-06

**Authors:** Tomoya Nakamura, Sofia Lavrenteva, Ikuya Murakami

**Affiliations:** Department of Psychology, The University of Tokyo, 7-3-1 Hongo, Bunkyo-ku, Tokyo 113-0033 Japan

**Keywords:** Psychology, Human behaviour, Neuroscience, Visual system

## Abstract

In visual backward masking paradigms, the visibility of a target is reduced using various kinds of mask stimuli presented immediately after the target. Four-dot masking is one such kind of backward masking, caused by four surrounding dots neither spatially adjacent nor similar to the target. Four-dot masking is often considered to involve object-level interferences. However, low-level contributions such as lateral inhibition and motion detection are also possible. To elucidate the loci of the underlying mechanism within the visual hierarchy, we compared the masking effect between monoptic and dichoptic viewing conditions. A target and a four-dot mask, which also served as a spatial cue to the target location, were presented to the same eye in monoptic viewing, whereas they were presented to different eyes in dichoptic viewing. Observers were then asked to discriminate the target shape. We found a significant decline in the correct response rate compared to the baseline condition in which the four-dot mask was not presented, and the masking effect was equivalent between the monoptic and dichoptic viewings. These results demonstrate that four-dot masking stems exclusively from processing within the binocular pathway.

## Introduction

Visual backward masking occurs when one visual stimulus (called a target) is rendered less visible by another stimulus (called a mask) delivered immediately after the target^1^. This phenomenon is classified into several kinds depending on the mask layout. For example, flashed uniform fields or various patterns such as random noise work as masks when they spatially overlap the target^2,3^. Masks that do not overlap the target can also exert backward masking. This kind of masking is called metacontrast masking^4^ and occurs when a mask has contours which are spatially adjacent to a target but do not overlap it. The effectiveness of both overlapping and non-overlapping masks depends on multiple factors including stimulus intensity^1,5^, mask size^6,7^, and spatial homogeneity^8,9^.

Besides, Kolers^5^ distinguished between Type-A and Type-B masking based on the temporal properties of backward masking. Type-A refers to a masking function that peaks when the stimulus onset asynchrony (SOA) between the target and mask is zero. Type-B function peaks when the SOA is positive, which is when the mask’s onset follows the target’s onset. To account for Type-B masking, a model of inter-channel inhibition based on the dual-channel theory has been proposed^10^. The dual-channel theory assumes a fast-transient channel with low spatial resolution and a slow-sustained channel with high spatial resolution. Type-B masking occurs because the sustained signals of a preceding target are interrupted by the transient signals of a subsequent mask^10^. This theory can also account for Type-A masking by assuming the integration of representations of the target and mask presented in temporal proximity.

Moreover, subsequent studies have reported that four dots surrounding but not adjacent to a target work as a Type-B mask^11,12^. This kind of masking is named four-dot masking due to its typical mask layout. When four-dot masking was initially reported, two features were highlighted. First, it is not much influenced by distance between a four-dot mask and a target^11,13–15^. Second, the effect increases at larger set sizes and decreases when a target appears to pop out or is pre-cued^13^, suggesting that four-dot masking involves a processing stage that is characterized by attentional modulation.

Considering these features, which imply the involvement of higher-level interferences in four-dot masking, Di Lollo et al.^13^ introduced the object substitution theory. Because the first feedforward input of the target is not a sufficient neural event for conscious perception to be established, higher-tier visual areas must iteratively send feedback reentrant signals to lower-tier visual areas to compare the higher-order representations with the current lower-order representations and to update the perceptual hypothesis about what the target is. If a signal from a subsequently presented mask newly comes into the system before recurrent processing is completed, the old target is substituted with the new mask; hence, “object substitution.” As a consequence, the system fails to create a target representation that is strong enough to be reportable. This theory also explains attentional modulation by assuming that attention to the target makes recurrent processing fast enough to be completed before the delivery of the mask, and can explain not only four-dot masking but also other classical maskings (e.g., pattern masking and classical metacontrast mentioned above)^16^.

However, other studies suspected that the recurrent processing assumed in the object substitution theory does not necessarily reflect the true neural mechanisms of four-dot masking. For example, Põder^17^ reinterpreted the object substitution theory^13^ with the notion of attentional gating^18^. In addition, a lateral inhibition model for metacontrast^19^ was extended to account for four-dot masking by using a neural network for response simulations^20^. Cross-correlations between the network’s responses to a target with a mask and without a mask, as an index of target visibility, successfully simulated the behaviour of four-dot masking. This model also predicted attentional modulation by assuming that attention to the target reduces the number of iterations of lateral inhibition.

Contrary to these models regarding the contribution of spatial attention, more recent studies have argued that distributed attention is not pivotal to four-dot masking. Under certain conditions, four-dot masking occurs when a target is fully attended to and foveated^21^. In addition, other studies found that contrary to earlier findings, four-dot masking is independent of stimulus set size^22,23^ and the size of an attended region^24^. These lines of evidence do not necessitate such attention-related accounts as object substitution and attentional gating but are more consistent with an account of motion correspondence between the target and mask. In this account, four-dot masking becomes effective when the target and mask cannot be individuated as separate objects and are bound as a single object because of the motion signal between them^15,25,26^. From the perspective of brain activities, repetitive transcranial magnetic stimulation delivered to area V5/MT+ or V1 led to the release from four-dot masking^27^, which also supports the contribution of motion processing. If motion correspondence is a key factor in four-dot masking, then it is worth investigating how motion sensing mechanisms may contribute to it.

Visual motion processing requires both monocular and binocular mechanisms^28^, but spatiotemporal correlations of luminance in the form of motion energy are predominantly sensed within the monocular pathway^29,30^, as also suggested by the partial interocular transfer of the static motion aftereffect^31^. If the occurrence of four-dot masking relies on energy-wise motion sensing mechanisms, its underlying mechanisms would then be predominantly monocular.

In the present study, we investigated whether the monocular pathway contributes to four-dot masking. We compared the extent of four-dot masking between monoptic viewing, in which the target and mask were presented to the same eye, and dichoptic viewing, in which they were presented to different eyes. During monoptic viewing, the target signal could be interrupted by the mask signal within either the monocular or binocular pathway, or within both pathways. However, masking-related processing within the monocular pathway would be impossible during dichoptic viewing because the signals of the target and mask must be carried through different monocular pathways from different eyes. The question posed above is viable because classical metacontrast still lacks conclusive evidence for the relative contributions of the monocular and binocular pathways^33–35^, and so does four-dot masking.

We aim to test three ideas. One is that four-dot masking exclusively involves the binocular pathway. This is presumably consistent with previous theories assuming high-level interactions such as object substitution because it is likely that the eye-of-origin information has been discarded before the stage of object-level representations. If this is the case, the strength of masking would be equivalent between the monoptic and dichoptic viewings. The second idea is that four-dot masking involves the binocular pathway but is also contributed to by monocular-level interactions to some extent. This may be consistent with the lateral inhibition model because lateral inhibition can occur within both the monocular and binocular pathways. In fact, during backward masking, lateral inhibitory signals from surrounding masks attenuated responses to a central target in LGN neurons, which are driven monocularly^32^. Based on this idea, monoptic masking is expected to be stronger than dichoptic masking. The last possible idea is that four-dot masking involves only early monocular spatiotemporal interactions that may be key to extracting motion correspondence. In this case, only monoptic masking would occur.

## Results

### The extent of four-dot masking does not differ between monoptic and dichoptic viewings

**Figure 1:**
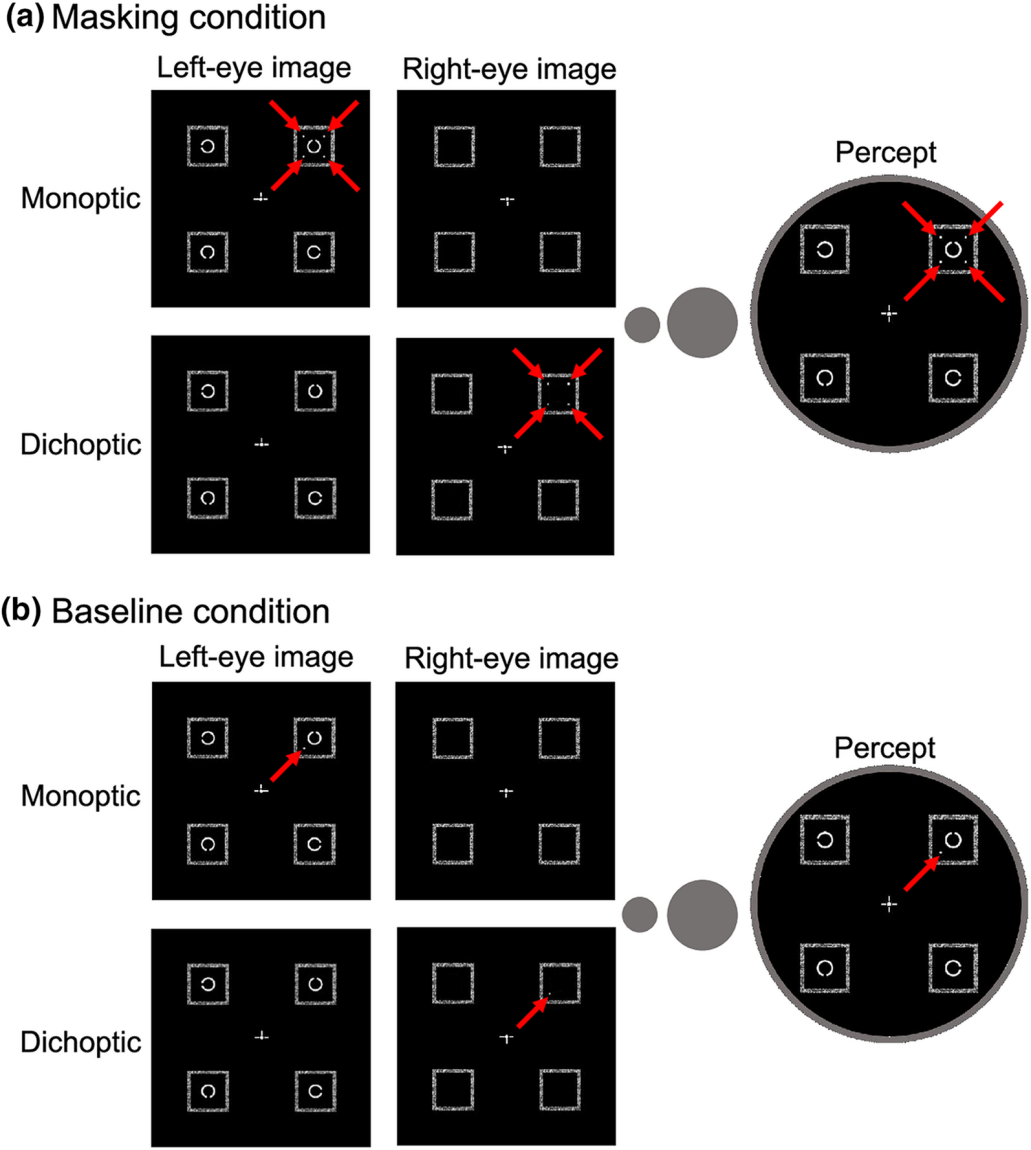
Snapshots of visual stimuli in the monoptic viewing (upper panels) and dichoptic viewing (lower panels). Observers binocularly fused the left-eye image and right-eye image to see a cyclopean view like the one depicted in the balloon labelled “Percept.” In the monoptic viewing condition, four Landolt-C-like stimuli (called Cs) were presented to one eye (the left eye here) and dots were presented to the same eye (the left eye). In the dichoptic viewing condition, four Cs were presented to one eye (the left eye) and dots were presented to the other eye (the right eye). Red arrows indicate the locations of the dots. (**a**) Masking condition: Four dots appeared around one of the Cs, indicating that this C was the target. (**b**) Baseline condition: Only the most proximal dot appeared, serving as a spatial cue to the target.

**Figure 2:**
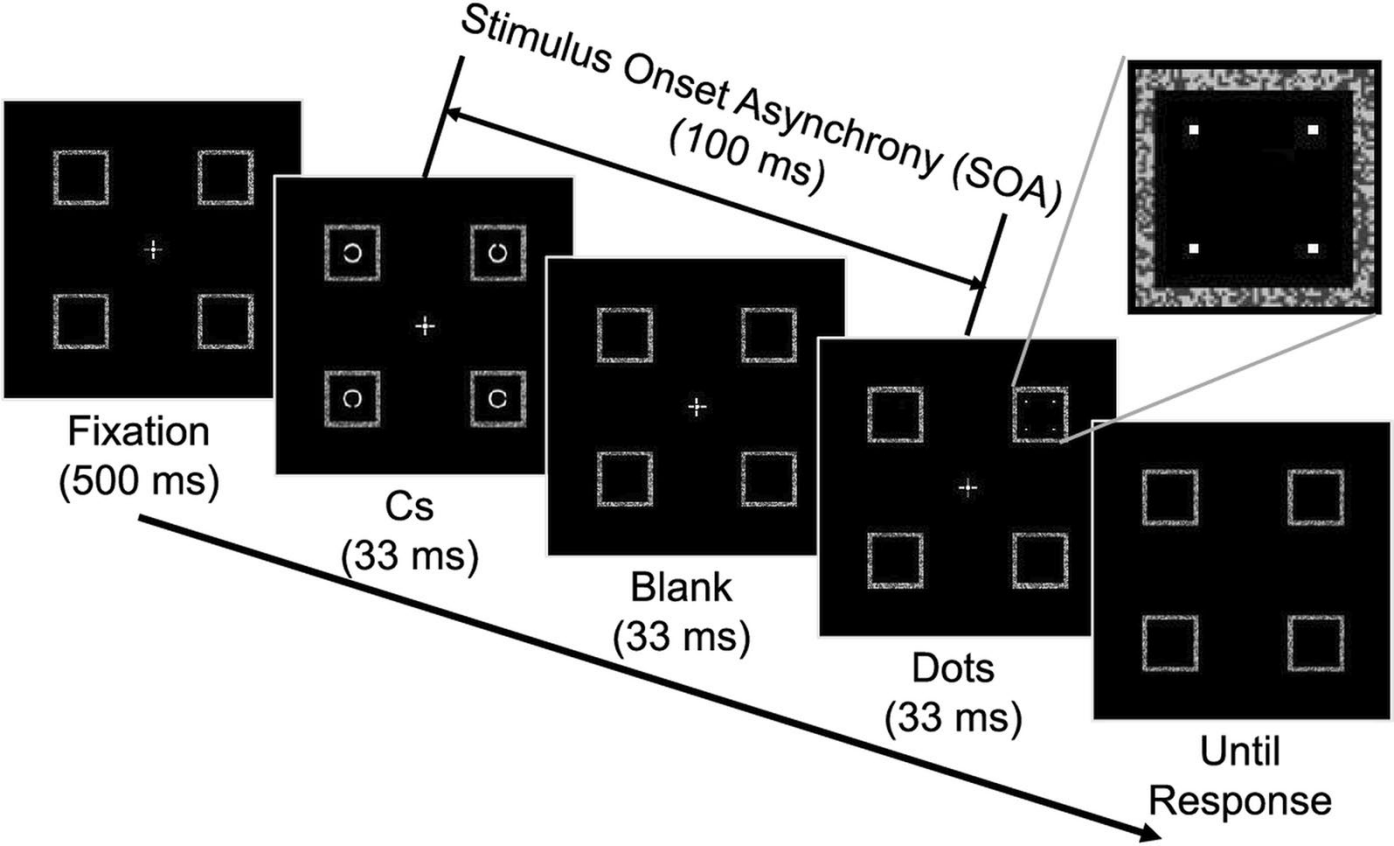
Schematic flow of cyclopean views in a trial at the SOA of 100 ms. The inset shows an enlarged view of the dots.

**Figure 3:**
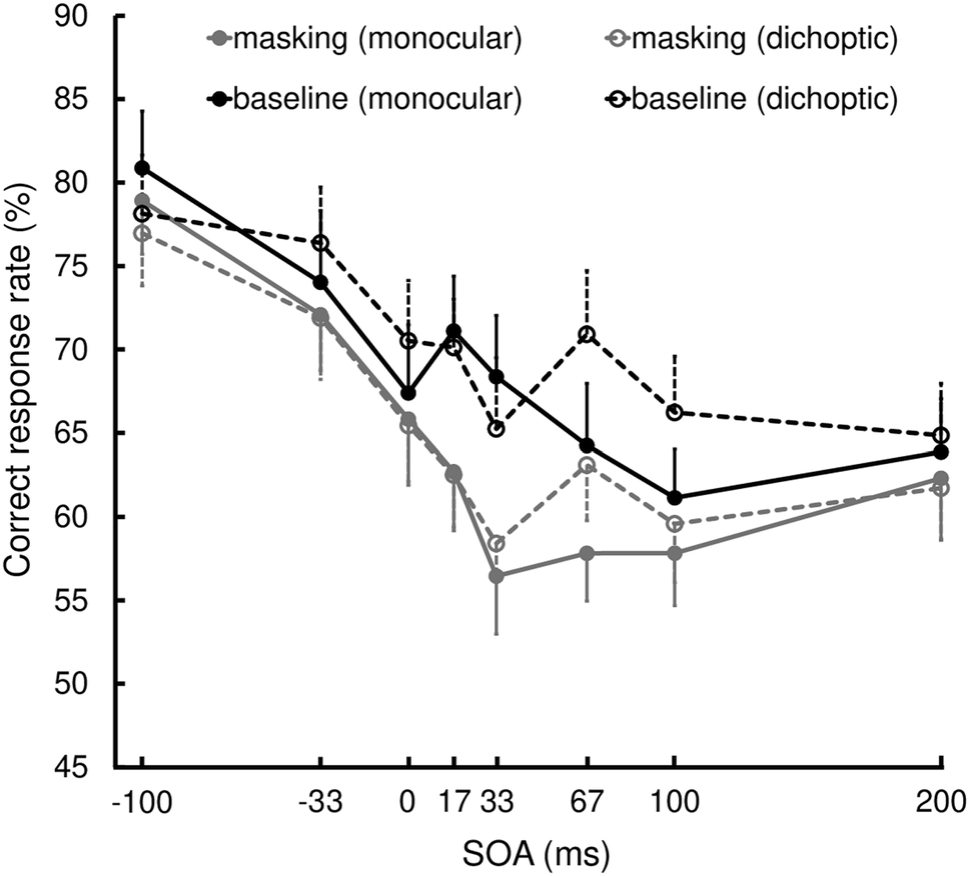
Interobserver mean (n = 16) of correct response rate in Experiment 1. The error bars indicate standard errors of the mean. The black and grey curves indicate the baseline and masking conditions, respectively. The solid curves indicate monoptic viewing, and the dashed curves indicate dichoptic viewing.

The purpose of Experiment 1 was to compare the extent of the four-dot masking effect between the monoptic and dichoptic viewings (Fig. 1). Four C-shaped stimuli were simultaneously presented, and one of them was either spatially cued as the target by four dots (the masking condition) or a single dot (the baseline condition). Observers were then asked to identify which part of the Landolt-C-like target was missing (Fig. 2). Figure 3 indicates the interobserver mean of the correct response rate as a function of SOA between the target and mask. The four-dot masking effect was defined as the correct response rate in the baseline condition minus that in the masking condition. We performed an 8 (SOA) by 2 (Mask: baseline and masking) by 2 (Eye: monoptic and dichoptic) repeated-measures analysis of variance (ANOVA), applying Holm’s step-down Bonferroni procedure^36^ to adjust the type-I errors.

The main effect of SOA was significant (*F*(7,105) = 45.15, *p* < 0.001, ${\eta }_{p}^{2}$ = 0.751), meaning that correct responses decreased as the SOA increased. The main effect of Mask was also significant (*F*(1,15) = 34.09, *p* < 0.001, ${\eta }_{p}^{2}$ = 0.695); the correct response rate was lower in the masking condition than in the baseline condition. This main effect provided evidence of masking in our experiment.

A significant interaction between SOA and Mask (*F* (7,105) = 2.90, *p* = 0.008, ${\eta }_{p}^{2}$ = 0.162) confirmed that the masking effect occurred within a limited temporal range, which is a signature of Type-B masking. In the post-hoc tests, we found significant simple effects of Mask at − 33, 17, 33, and 67 ms SOA (*F* (1,15) = 4.65, *p* = 0.048, ${\eta }_{p}^{2}$ = 0.237; *F* (1,15) = 33.25, *p* < 0.001, ${\eta }_{p}^{2}$ = 0.689;* F* (1,15) = 31.70, *p* < 0.001, ${\eta }_{p}^{2}$ = 0.679;* F* (1,15) = 14.90, *p* = 0.002, ${\eta }_{p}^{2}$ = 0.498), but not at − 100, 0, 100, and 200 ms SOA (*F* (1,15) = 1.01, *p* = 0.330, ${\eta }_{p}^{2}$ = 0.063; *F* (1,15) = 3.38, *p* = 0.086, ${\eta }_{p}^{2}$ = 0.184;* F* (1,15) = 4.32, *p* = 0.055, ${\eta }_{p}^{2}$ = 0.224;* F* (1,15) = 1.41, *p* = 0.253, ${\eta }_{p}^{2}$ = 0.086).

However, there was no significant interaction between Mask and Eye (*F* (1,15) = 0.22, *p* = 0.647, ${\eta }_{p}^{2}$ = 0.014), and there was no significant second-order interaction (*F* (7,105) = 0.51, *p* = 0.823, ${\eta }_{p}^{2}$ = 0.033). Therefore, the masking effect was equally vigorous in both the monoptic and dichoptic viewings.

Contrary to our expectations, a significant interaction between SOA and Eye was also found (*F* (7,105) = 2.47, *p* = 0.022, ${\eta }_{p}^{2}$ = 0.142), while the main effect of Eye was not significant (*F* (1,15) = 2.01, *p* = 0.176, ${\eta }_{p}^{2}$ = 0.118). In the post-hoc tests, we found significant simple effects of Eye at 67 and 100 ms SOA (*F* (1,15) = 8.49, *p* = 0.011, ${\eta }_{p}^{2}$ = 0.361; *F* (1,15) = 13.23, *p* = 0.002, ${\eta }_{p}^{2}$ = 0.469), but not at − 100, − 33, 0, 17, 33, and 200 ms (*F* (1,15) = 2.00, *p* = 0.178, ${\eta }_{p}^{2}$ = 0.118; *F* (1,15) = 0.46, *p* = 0.508, ${\eta }_{p}^{2}$ = 0.030;* F* (1,15) = 0.46, *p* = 0.509, ${\eta }_{p}^{2}$ = 0.030;* F* (1,15) = 0.14, *p* = 0.718, ${\eta }_{p}^{2}$ = 0.009;* F* (1,15) = 0.11, *p* = 0.747, ${\eta }_{p}^{2}$ = 0.007;* F* (1,15) = 0.01, *p* = 0.918, ${\eta }_{p}^{2}$ < 0.001). Thus, the dichoptic performance was significantly better than the monoptic performance when the SOA was 67–100 ms.

To recap, we obtained three findings. First, the correct response rate declined as the SOA increased. This seems natural because the dots served not only as a mask but also as a sole cue to the target in the present paradigm. Negative SOAs meant that the observer was informed of the location of the impending target, whereas positive SOAs meant that the cue to the target was delivered after the four Cs physically disappeared, only dwelling in some form of visual memory, which would soon become unavailable even if no masking took place.

Second, four-dot masking occurred when the SOA was − 33 and 17–67 ms, while the masking effect was nearly significant when the SOA was 0 and 100 ms. The range of effective SOAs was consistent with previous reports of four-dot masking (e.g., − 45 to 90 ms SOA)^11^. It is worth noting that these characteristics were equally observed across the monoptic and dichoptic viewings. The equivalence of the masking effect irrespective of which eye was stimulated by the target and mask indicates that four-dot masking depends on the processing level within the binocular pathway in which the eye-of-origin information has been lost.

**Figure 4:**
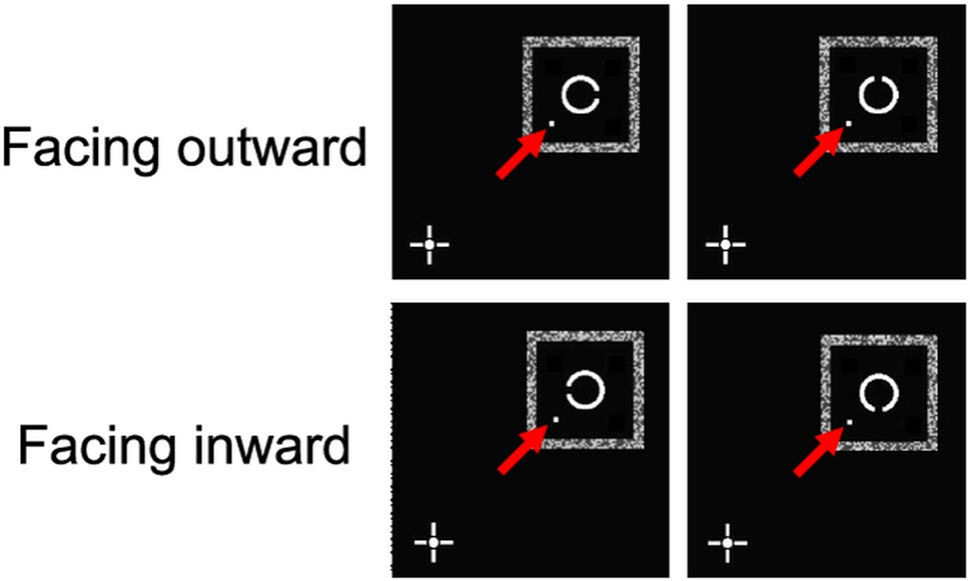
Spatial configurations of the target gap and the dot in the baseline condition when the target was presented in the first quadrant of the display. In the upper panels, the target gap is facing outward, and the distance between the gap and dot is larger. In the lower panels, the target gap is facing inward and the distance is smaller. Red arrows indicate the locations of the dots.

Third, at larger SOAs (67 and 100 ms), the dichoptic performance was better than the monoptic performance in both the masking and baseline conditions. This eye-specific effect needs an explanation of its own because the effect, seen even in the baseline condition, must be logically independent of four-dot masking. Our preliminary analysis suggests that the target was rendered less identifiable with the presence of a single dot as a cue to the target location in the baseline condition. The performance was about 7% worse when the target gap was facing inward than when it was facing outward, regardless of the SOA. For example, consider the target presented to the first quadrant (Fig. 4). In the two upper panels, the target gap is facing outward (right and top), and the distance between the gap and the dot is relatively large. In contrast, in the two lower panels, the target is facing inward (left and bottom), and the distance is relatively small. Consequently, the small distance between the gap and the baseline dot could have decreased the discrimination performance in an eye-specific manner; such interference might have been stronger when the target and dot were delivered to the same eye. Therefore, in Experiment 2, we sought to confirm whether the four-dot masking effect was maintained as it was in Experiment 1, whereas the eye-specific effect would disappear by reducing potential dot-gap interference as much as possible.

### The results are replicable even when the spatial cue is independent of the mask

**Figure 5:**
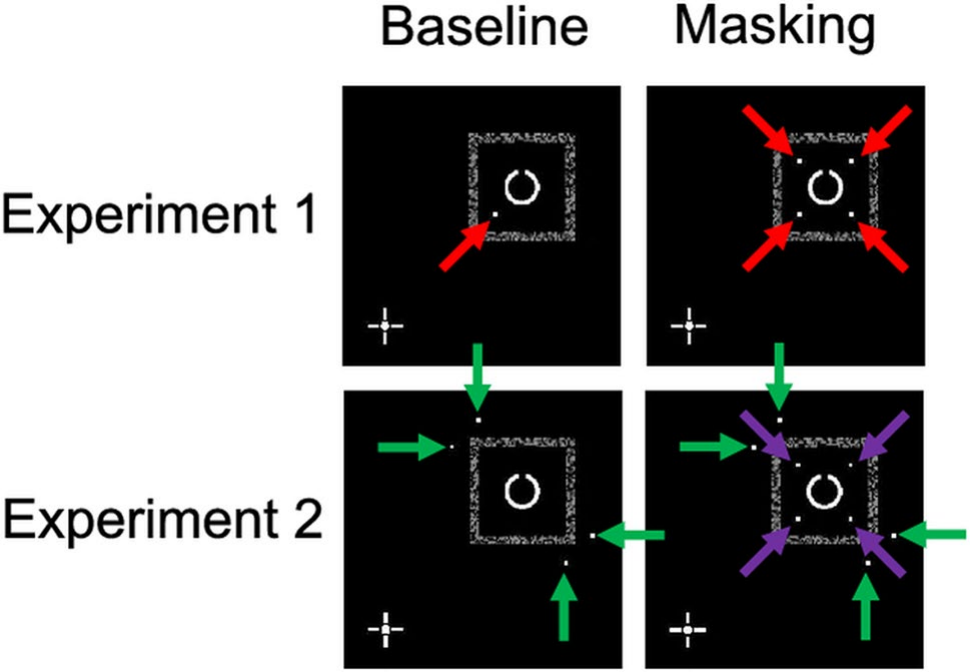
Configurations of the target and dots in Experiment 1 (upper panel) and Experiment 2 (lower panel). In this example, the target is presented in the first quadrant of the display. The left and right panels indicate the baseline and masking conditions, respectively. Red arrows indicate the locations of the dots in Experiment 1. The green and purple arrows indicate the locations of the dots as a cue and mask in Experiment 2, respectively.

**Figure 6:**
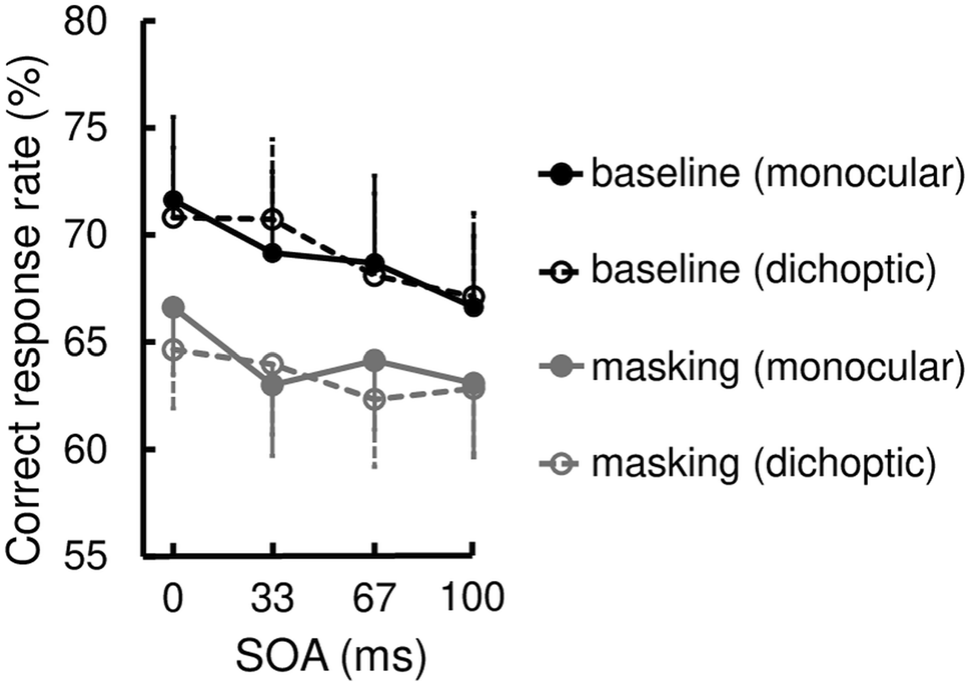
Interobserver mean (n = 19) of correct response rate in Experiment 2. The error bars indicate standard errors of the mean. The black and grey curves indicate the baseline and masking conditions, respectively. The solid curves indicate monoptic viewing, and the dashed curves indicate dichoptic viewing.

The purpose of Experiment 2 was to obtain a clearer picture of four-dot masking in the monoptic and dichoptic viewings with minimal involvement of dot-gap interference. As a single dot in Experiment 1 was deemed to have played an interferential role, it was replaced by four dots presented outside the placeholder (Fig. 5), and we examined a narrower range of SOA than we did in Experiment 1, but otherwise the methods were essentially identical to those of Experiment 1. Figure 6 indicates the interobserver mean of the correct response rate as a function of SOA. We performed a 4 (SOA) by 2 (Mask: baseline and masking) by 2 (Eye: monoptic and dichoptic) repeated-measures ANOVA applying Holm’s step-down Bonferroni procedure^36^.

The main effect of SOA was significant (*F*(3,54) = 6.30, *p* = 0.001, ${\eta }_{p}^{2}$ = 0.259), meaning that the correct response rate decreased as the SOA increased, which is consistent with the results of Experiment 1.

The main effect of Mask was also significant (*F*(1,18) = 13.64, *p* = 0.002, ${\eta }_{p}^{2}$ = 0.431); the correct response rate was lower in the masking condition than in the baseline condition. Thus, we replicated the four-dot masking effect. This time, the interaction between SOA and Mask was not significant, unlike in Experiment 1 (*F*(3,54) = 0.76, *p* = 0.523, ${\eta }_{p}^{2}$ = 0.040), which means that the masking effect was significant at all SOAs. This was expected because only a critical range of SOA needed for four-dot masking to occur maximally was chosen in Experiment 2.

Unlike these two replicated effects, no pertinent effects to the factor Eye were significant (main effect of Eye: *F*(1,18) = 0.23, *p* = 0.638, ${\eta }_{p}^{2}$ = 0.013; SOA × Eye interaction: *F* (3,54) = 1.08, *p* = 0.364, ${\eta }_{p}^{2}$ = 0.057; Mask × Eye interaction: *F* (1,18) = 0.49, *p* = 0.494, ${\eta }_{p}^{2}$ = 0.026; SOA × Mask × Eye interaction: *F* (3,54) = 0.01, *p* = 0.998, ${\eta }_{p}^{2}$ < 0.001). Thus, we replicated the equivalence of the masking effect between the monoptic and dichoptic viewings observed in Experiment 1. In addition, the eye-specific effect observed in Experiment 1 disappeared when the decrease in performance in the baseline condition was minimized. These results corroborate that the eye-specific effect observed in Experiment 1 is irrelevant to four-dot masking.

## Discussion

Through the two experiments, we came up with three findings. First, we found a considerable decline in overall performance with increasing SOA, as also found by Enns^37^. This is expected because in our paradigm, observers had no way to figure out which of the four Cs was to be the target until the dots were delivered. Accordingly, the main effect of SOA should be mainly attributed to the memory loss of the target shape.

Second, when the SOA was − 33 and 17–67 ms (Experiment 1) and 0–100 ms (Experiment 2), the performance in the masking condition was lower than that in the baseline condition, indicating the existence of four-dot masking. The range of effective SOAs for four-dot masking is comparable to the data found in previous studies (e.g., − 45 to 90 ms SOA)^11^, thus not only backward but also forward and simultaneous masking effects were seen. According to the definition by Enns^37^, we defined the masking effect as the decline of performance in the masking condition compared with the performance in the baseline condition. In an experiment in Enns’s study^37^, which applied an experimental paradigm similar to ours, the extent of the masking effect was approximately 20% at a set size of 4, which is the same set size as ours. Compared with Enns’s, our experiments exhibited smaller masking effects (< 12% in Experiment 1 and < 7% in Experiment 2). However, Argyropoulos et al. reported that four-dot masking was small when the overall performance was far from both the ceiling and floor^22^; in this aforementioned study, a comparable extent of masking was found when a paradigm similar to ours was used.

Third, the extent of the masking effect did not significantly differ between the monoptic and dichoptic viewings at any SOA. This equivalence is considered as psychophysical evidence indicating that four-dot masking exclusively involves the binocular pathway. This conclusion may be consistent with previous theories assuming high-level interactions such as object substitution^13^ because the higher processing stages required for object-level representations would have progressively fewer monocular neurons^38^. As for lateral inhibition^20^, our findings are incompatible with lateral inhibition that occurs within the monocular pathway including the retina, the lateral geniculate nucleus (LGN), and the monocular cortical neurons^32,39,40^, where signals from the same eye-of-origin laterally inhibit each other, making dichoptic masking unlikely^41^. Nevertheless, the lateral inhibition model is still viable if lateral inhibition is allowed to occur across signals originating from different eyes.

There are several layouts of mask exerting backward masking, and each kind of masking may receive certain monocular and binocular contributions. Thus, it is worth comparing the contribution of the monocular pathway among previously reported kinds of backward masking. One of the simplest kinds of backward masking is masking by a flashed uniform field; this never occurs in dichoptic viewing when the target and mask are presented to different eyes^2,42,43^, suggesting that masking by flash is derived only from monocular mechanisms. Next, in a typical pattern-masking paradigm, an overlapping pattern whose visual structure is similar to the target shape exerts a masking effect even in dichoptic viewing, although the effect is generally weaker than that in monoptic viewing^42,43^. Thus, it follows that masking by overlapping patterns is partially contributed to by monocular mechanisms. As for metacontrast, dichoptic masking effects were observed in most studies^33–35^. Schiller and Smith^34^ found an even stronger effect in dichoptic viewing than in monoptic viewing at small SOAs, suggesting a possible contribution of binocular rivalry^44^ as the origin of the devastating effect in dichoptic viewing. Weisstein^35^ found that in dichoptic viewing, the range of SOA for the metacontrast masking effect was broader and the peak SOA, where masking was most effective, was smaller than in monoptic viewing. Probably due to the presence of adjacent contours, interocular suppressions could have resulted in different metacontrast masking functions between the monoptic and dichoptic viewings. Overall, previous knowledge suggests that dichoptic effects should be accounted for not only by interocular suppression artefacts but also by interactions at a binocular level^45^. However, unequivocal demonstrations of equivalence between monoptic and dichoptic effects are still lacking. Compared to these classical masking effects, the present study demonstrates masking at a completely binocular level without any possible concern of interocular suppression because the mask and the target were too widely separated from each other to evoke suppression even if they were presented statically. Our observation does not contradict some studies suggesting that four-dot masking largely involves local image-level interferences as in classical accounts of metacontrast rather than global object-level interferences^46,47^. The idea that metacontrast and four-dot masking may share the same low-level mechanism is also consistent with recent studies showing that both kinds are immune to attention^21–24,48,49^.

However, even though interferences may be local, they do not pertain to local spatiotemporal interactions involved in monocular sensing of motion energy. The potential contribution of motion sensing mechanisms to four-dot masking has been discussed in some previous studies^15,25–27^. These studies point out that object continuity between a target and a mask based on motion correspondence is vital to object substitution. In contrast, our results demonstrated that low-level monocular motion energy computation does not contribute to four-dot masking. If motion correspondence based on visual motion sensing is integral to four-dot masking, then the underlying mechanisms should be capable of interocular computations of motion correspondence^28,30^.

In the same context, we used SOA as an independent variable to control the temporal relationship between the target and mask^11,26,37^ as it is one of the most critical factors to be able to perceive motion^50^. Many recent studies on four-dot masking preferred using a common onset paradigm, in which mask duration is varied while the onsets of the target and mask are fixed^13,21–24^. Although this paradigm may also involve motion perception, this is not the ideal condition to activate the underlying mechanisms of motion detection^51^; thus, in the present study, we set a stringent condition to ensure the absence of contribution of conventional motion detectors that must become optimally activated at critical SOAs. Besides, using the SOA paradigm enabled us to compare our results with those of previous studies investigating more classical kinds of backward masking mentioned above since most of them adopted the SOA paradigm^33–35,42,43^.

One thing that needs explanation in the data from Experiment 1 is that when the SOA was 67–100 ms, the dichoptic performance became higher than the monoptic performance in both the baseline and masking conditions. This eye-specific effect is irrelevant to four-dot masking, but it is interesting to ask what kind of process is responsible for this effect. One possibility is worth mentioning in reference to the results of Experiments 1 and 2. The largest difference between the two experiments was whether the spatial cue to the target location in the baseline condition was also a part of the four-dot mask in the masking condition. In Experiment 1, the most proximal dot was sufficient for observers to figure out the target location, so that they might have some attentional set to maintain focus on the most proximal dot. This might have strengthened the low-level monocular suppression of the target by the presence of the most proximal dot in both the baseline and masking conditions. In fact, when we abandoned the use of the most proximal dot as a spatial cue to the target location and presented a cue of a new shape, namely the outer four dots, at sufficiently separated locations from the target (Fig. 5), the eye-specific effect observed in Experiment 1 disappeared in Experiment 2. In any case, we are still unsure as to what kind of eye-specific mechanisms are really involved, but identifying the real cause is outside the scope of the present study. It is important to note though that this eye-specific effect is logically independent of four-dot masking because this effect occurred in both the baseline and masking conditions in Experiment 1 and disappeared in both the baseline and masking conditions in Experiment 2.

In summary, we compared the four-dot masking effect between the monoptic and dichoptic viewings and obtained an equivalent masking effect. Therefore, we conclude that four-dot masking exclusively involves the binocular pathway. The lack of evidence of any contributions by the monocular pathway is compatible with some previous models of four-dot masking assuming object-level interference, but it refuted the idea that object updating based on early motion energy computation is essential to four-dot masking.

## Methods

### General methods

In total, 32 adults with normal or corrected-to-normal visual acuity participated. They were naïve to the purpose of the study, except for two who were the first and second authors. All participants provided their written informed consent. The experiments were conducted in accordance with the Declaration of Helsinki and were approved by the ethics committee of the Graduate School of Humanities and Sociology at the University of Tokyo.

Computer programs were written in MATLAB R2018a (MathWorks, Natick, MA) with the Psychophysics Toolbox extension version 3.0.14^52–54^ and were run on a computer (Apple MacPro Late 2013). Visual stimuli were displayed on a head-mounted display (Sony HMZ-T3) with a spatial resolution of 1,280 × 720 pixels and a refresh rate of 60 Hz. The head-mounted display was used as an optimal kind of stereoscope for the experimental purpose because it had one organic-light-emitting-diode display for each eye with no possibility of crosstalk in principle.

### Procedure of Experiment 1: comparison of the masking effect between the monoptic and dichoptic viewings

16 observers (5 females) participated in the study. The background (subtending 45° horizontally and 25° vertically) was uniformly black. We used a set of Nonius lines for the vertical components of a 0.7° × 0.7° fixation cross. Thus, two vertical lines above and below the central point were presented to the left and right eyes, respectively, whereas two horizontal lines to the left and right of the central point were presented to both eyes. Each observer was instructed to adjust the inter-display distance of the goggles so that the vertical lines would appear aligned when their virtual images were roughly located at reading distance.

Throughout each trial, four placeholders were binocularly presented at the same eccentricity, which is 7.7°. These were square frames (outer sides 4.2 × 4.2°, inner sides 3.9 × 3.9°) composed of fine random-dot patterns that aided the maintenance of binocular fusion. Inside the placeholders, we presented two kinds of stimuli. One was a white Landolt-C-like stimulus (radius 0.7°, ring width 0.2°) with a 0.3°-wide gap on the top, bottom, left, or right of the ring, hereinafter referred to as stimulus “C.” Four such Cs were simultaneously presented, each at the centre of each placeholder. The other stimulus was four small white dots (0.4° × 0.4° each) configuring a square (2.1° × 2.1°) shape concentrically surrounding one of the four Cs.

In the masking condition (Fig. 1a), the “target” was defined as the C accompanied by these four dots, whereas all other Cs were distractors. The presence of the four dots was expected to reduce the target visibility given that four-dot masking occurred. Thus, the four dots served as a spatial cue to the target as well as a “mask.” All Cs were presented to one eye, either the left or right, and the dots were presented to the same eye (monoptic viewing) or the other eye (dichoptic viewing).

In the baseline condition (Fig. 1b), a single dot instead of four dots was used as a cue to the target^37^. This dot was the most proximal of all four dots in the masking condition and defined the target just as the four dots did, but it was not expected to mask the target, unlike in the case of the four-dot masking.

We used an 8 (SOA: − 100, − 33, 0, 17, 33, 67, 100, and 200 ms) by 2 (Mask: baseline and masking) by 2 (Eye: monoptic and dichoptic) factorial design. The eye to which the target was presented was randomly chosen in each trial. The positive SOAs denote that the Cs were followed by the dots. The masking and baseline conditions were tested in separate sessions. There were 512 (8 SOAs × 2 Eye conditions × 32 repetitions) trials for each of the masking and baseline conditions. These trials were carried out in four sessions in a pseudo-random order. As such, we conducted four masking and baseline sessions in an interleaved manner. The order of the sessions was counterbalanced across observers.

At the beginning of each trial, the fixation cross and placeholders were presented for 500 ms, followed by an additional delivery of the Cs and dots, for a duration of 33 ms each, with an SOA between them. For example, when the SOA was 100 ms, the Cs were initially presented for 33 ms, the placeholders were left blank for the next 67 ms, and the dots were finally presented for 33 ms. When the SOA was − 100 ms, the dots were initially presented for 33 ms, the placeholders were left blank for the next 67 ms, and the Cs were finally presented for 33 ms. When the SOA was 0 ms, the Cs and dots appeared and disappeared altogether. Figure 2 shows the time course of a single trial when the SOA was 100 ms. Regardless of the SOA, the fixation cross disappeared together with the last offset of the Cs and dots, which signalled the observer to indicate which part (top, bottom, left, or right) of the target was missing by pressing one of four computer keys. The observer’s self-paced key pressing triggered a beep sound, which did not provide any informative feedback except for signalling the start of the next trial 2.5 s later.

### Procedure of Experiment 2: replication of Experiment 1 without interferences at the baseline

The purpose of Experiment 2 was to examine whether the equivalence of the masking effect between the monoptic and dichoptic viewings found in Experiment 1 was replicable even when the decrease in baseline performance possibly originating from dot-gap interference was minimized. The procedures were identical except for the SOA levels, the number of trial repetitions, and the arrangements of dots.

20 observers (4 females) participated in the study. Four of them were observers from Experiment 1. One observer was excluded before completing the experiment as he was unable to keep fusing the placeholders. We used a 4 (SOA: 0, 33, 67, and 100 ms) by 2 (Mask: baseline and masking) by 2 (Eye: monoptic and dichoptic) factorial design. There were 512 (4 SOAs × 2 Eye conditions × 64 repetitions) trials for each of the masking and baseline conditions.

We changed the arrangements of dots from Experiment 1 in an attempt to remove potential interference that should be irrelevant to four-dot masking. Figure 5 shows the difference in stimulus arrangement between Experiments 1 and 2. In the baseline condition, four dots outside the placeholder were used in Experiment 2 instead of a single dot in Experiment 1; the centroid of the four dots was the same as that of the target. By doing this, we intended to maintain attentional focus on the target location at the moment when the dots were presented. In addition, we expected that these very distant dots would neither locally interfere with the target nor become perceptually grouped with the target. In the masking condition, we further added four dots inside the placeholder, and these four dots were the same as those used in Experiment 1. By doing this, the outer four dots consistently served as a spatial cue to the target location, whereas the inner four dots served as a mask in four-dot masking. The outer four dots and the inner four dots were presented simultaneously.

## Data Availability

The dataset and program code of the present study are available from the corresponding author upon request.
